# Grammatical Class Effects Across Impaired Child and Adult Populations

**DOI:** 10.3389/fpsyg.2015.01670

**Published:** 2015-11-17

**Authors:** Maria Kambanaros, Kleanthes K. Grohmann

**Affiliations:** ^1^Department of Rehabilitation Sciences, Cyprus University of TechnologyLimassol, Cyprus; ^2^Department of English Studies, University of CyprusNicosia, Cyprus; ^3^Cyprus Acquisition TeamNicosia, Cyprus

**Keywords:** anomia, aphasia, lexical retrieval, multiple sclerosis (MS), noun–verb dissociation, picture naming, schizophrenia-spectrum disorder (SCZ), specific language impairment (SLI)

## Abstract

The aims of this study are to compare quantitative and qualitative differences for noun/verb retrieval across language-impaired groups, examine naming errors with reference to psycholinguistic models of word processing, and shed light on the nature of the naming deficit as well as determine relevant group commonalities and differences. This includes an attempt to establish whether error types differentiate language-impaired children from adults, to determine effects of psycholinguistic variables on naming accuracies, and to link the results to genetic mechanisms and/or neural circuitry in the brain. A total of 89 (language-)impaired participants took part in this report: 24 adults with acquired aphasia, 20 adults with schizophrenia-spectrum disorder, 31 adults with relapsing-remitting multiple sclerosis, and 14 children with specific language impairment. The results of simultaneous multiple regression analyses for the errors in verb naming compared to the psycholinguistic variables for all language-impaired groups are reported and discussed in relation to models of lexical processing.

This discussion will lead to considerations of genetic and/or neurobiological underpinnings:

1. Presence of the noun–verb dissociation in focal and non-focal brain impairment make localization theories redundant, but support for wider neural network involvement.
2. The patterns reported cannot be reduced to any one level of language processing, suggesting multiple interactions at different levels (e.g., receptive vs. expressive language abilities).
3. Semantic-conceptual properties constrain syntactic properties with implications for phonological word form retrieval.
4. Competition needs to be resolved at both conceptual and phonological levels of representation.

Moreover, this study may provide a cross-pathological baseline that can be probed further with respect to recent suggestions concerning a reconsideration of open- vs. closed-class items, according to which verbs may actually fall into the latter rather than the standardly received former class.

## Aims and background

There is a wealth of research spanning half a century on grammatical class effects exemplified by the noun–verb dissociation in language production. Evidence over the years for a noun superiority over verbs, or possibly vice versa, in spoken naming has come from a wide variety of sources such as the following, non-exhaustive list:

- aphasia resulting from stroke classified as either Broca's aphasia (agrammatism) or anomic aphasia (e.g., Jonkers and Bastiaanse, 1998; Luzzatti et al., 2002; Kambanaros, 2008; Franco et al., 2012; Franco, 2014);
- left subcortical lesions and concomitant anomia (Kambanaros and van Steenbrugge, 2006);
- non-focal or diffuse brain pathology such as dementia (Miozzo et al., 1994; Robinson et al., 1996; White-Devine et al., 1996; Bushell and Martin, 1997; Silveri and di Betta, 1997; Cappa et al., 1998), Alzheimer's disease (Druks et al., 2006), and primary progressive aphasia (Thompson et al., 2012);
- motor-related neurological diseased groups such as Parkinson's disease (see Herrera and Cuetos, 2012 for an update), progressive supranuclear palsy (Daniele et al., 2013), and corticobasal degeneration (Silveri and Ciccarelli, 2007);
- psychiatric disorders, including schizophrenia (Kambanaros et al., 2010);
- developmental language disorders, most prominently specific language impairment, henceforth SLI (Sheng and McGregor, 2010; Kambanaros et al., 2013a,b);
- genetic syndromes such as Williams syndrome (Thomas et al., 2006);
- bi- and multilingual individuals who show the noun–verb dissociation in all their spoken languages, such as multilingual patients with aphasia following stroke (Kambanaros and van Steenbrugge, 2006; Kambanaros, 2009b; Faroqi-Shah and Waked, 2010), primary progressive aphasia (Hernández et al., 2008), multiple sclerosis (Calabria et al., 2014), and multilingual children with SLI (Kambanaros et al., 2013a, 2014);
- modality-specific dissociations, that is, disproportionate impairments in naming words from one grammatical class (nouns or verbs) in the spoken or written modality only (Caramazza and Hillis, 1991; Hillis and Caramazza, 1995; Rapp and Caramazza, 1998, 2002; Kambanaros, 2015);
- cross-linguistic findings of the noun–verb dissociation for languages with different underlying morphological characteristics, including minimally inflected languages such as English, highly inflected languages such as Greek, and languages with no morphology or inflection such as Chinese (for extensive review and references, e.g., Kambanaros, 2009b; Kemmerer, 2014).

Historically, the robust evidence for the noun–verb dissociation based on selective impairments of grammatical categories in spoken naming seen in patients with defined lesions was considered in relation to damage to the distinct underlying neural substrates for nouns and verbs (for a detailed review of past and present research, see Vigliocco et al., 2011). Specifically, in cases of double dissociations (see studies reported in Kambanaros, 2009b), damage to the left prefrontal cortex or the motor-processing areas was typically associated with verb deficits (e.g., Broca's aphasia), and damage to the left posterior areas, in particular the temporal lobe or visual-object processing regions, with noun deficits (e.g., Wernicke's aphasia). However, recent, more sophisticated analyses of past neuroimaging evidence has revealed that verb and noun processing sites are not spatially segregated as originally considered, but encompass areas that are overlapping and intertwined in a more distributed left fronto-temporal-parietal network (for a recent update, see Crepaldi et al., 2013).

There is a plethora of cross-linguistic evidence that the ability to produce verbs and nouns can be differentially affected in aphasia (Kambanaros, 2009b), although no consistent patterns have been so far identified to suggest discrete links between lesion site and verb/noun processing differences (Arévalo et al., 2011). Nevertheless, such disproportionate impairments have guided hypotheses about the organization of linguistic representations in the brain.

Understanding how brain damage disrupts language production focusing on the noun–verb dissociation in spoken naming contributes, on the one hand, to the fine-tuning of theories related to the representation of semantic knowledge (Bird et al., 2000), and, on the other, to the underlying mechanisms supporting lexical access (Levelt et al., 1999). What is now clear is that word-retrieval deficits for nouns and verbs are evident in individuals with different patterns of brain damage beyond circumscribed focal lesions (see groups reported in (i)–(x) above). The present article reports on studies investigating noun and verb word-retrieval differences for different language-impaired (LI) groups on the same measure, the Greek Object and Action Test (GOAT), a tool designed for the purpose of measuring grammatical word class processing and retrieval (Kambanaros, 2003). The specific emphasis is on spoken naming accuracy of nouns and verbs by the following clinical groups:

- adult groups with acquired aphasia (Broca's aphasia and anomic aphasia),
- individuals with relapsing–remitting multiple sclerosis (RRMS),
- individuals diagnosed with schizophrenia-spectrum disorder (SCZ) and
- school-aged children with specific language impairment (SLI).

The clinical characteristics of each LI group are reported in Table 1.

**Table 1:** **Clinical characteristics of participating LI groups**.

**LI Group**	**Onset**	**Etiology**	**Brain pathology**	**NVIQ**	**Word compr**.	**Expressive language**	**Speech output**	**Group description**
BA	Acquired	Focal lesion	Focal lesion in Broca's area	Normal	Preserved	Syntactic deficits	Non-fluent	Homogenous
AA	Acquired	Focal lesion	Focal lesion in the parietal or temporal lobes	Normal	Preserved	Anomia	Fluent	Homogenous
SCZ	Acquired	Non-focal	Frontal & temporal lobes, prefrontal cortex, medial temporal lobes, thalamus	Normal	Preserved	Mild receptive & expressive language deficits, if any language involvement	Fluent	Homogenous
RRMS	Acquired	Non-focal	Damage to language-dedicated networks in both the IFG and MFG and the ROL	Normal	Preserved	Preserved	Fluent	Homogenous
SLI (ch.)	Developmental	Unknown (candidate genes?)	Largely intact brain but abnormalities of brain structures in left frontal BA 44, premotor, basal ganglia (caudate) circuits, cerebellum, inferior parietal cortex, superior temporal cortex	Normal	Preserved	Deficits in grammar and lexicon	Fluent	Heterogeneous

*LI, language-impaired; NVIQ, non-verbal intelligence quotient; compr., comprehension; BA, Broca's aphasia; AA, anomic aphasia; SCZ, schizophrenia; RRMS, relapsing remitting multiple sclerosis; SLI, specific language impairment; ch., children; IFG, inferior frontal gyrus; MFG, middle frontal gyrus; ROL, left Rolandic operculum*.

The GOAT is a picture-based measure of noun and verb comprehension and production. Although not without criticism for not being considered ecological (see Herbert et al., 2008 for an explanation), confrontation picture naming is the most commonly used task for the assessment of the noun–verb dissociation in spoken production (Kambanaros, 2008, 2010) as it taps into an individual's knowledge about a target word. In fact, naming a picture involves multiple stages, each with its own specific activation patterns in the brain (see Indefrey, 2007, 2011). First comes the *pre-semantic* stage of processing where, upon seeing a picture, the speaker recognizes the concept as a noun/object (e.g., spade for a picture of a spade) or as a verb/event (e.g., dig(ging) for a picture of someone digging). This is followed by the *semantic stage* where a set of meaning-related lexical items are activated. For example, seeing a picture of the action “digging” could also activate actions such as “raking” or “sweeping.” Next, a name is retrieved after the concept is distinguished from visually and semantically similar items. This is called the *lexical-semantic* stage. Finally, the phonological form of the target word is made available. For spoken naming, representation of the sound of the word is activated in the phonological output lexicon, then stored in the phonological assembly buffer from where instructions are given to the sensory-motor system to coordinate and produce speech sounds (e.g., /sped/ or /′dıg(ıη)/). Picture naming is considered a task with low selection demand (i.e., one specific word is retrieved from memory), given that there is a single dominant response, and usually interpreted as a general (low-level) language outcome measure in acquired language impairments (Barwood and Murdoch, 2013).

Moreover, major lexical-semantic variables of the noun and verb stimuli influence performance on lexical retrieval tasks for both adults with acquired language impairments (Druks et al., 2006; Masterson et al., 2007; Rodriguez-Ferreiro et al., 2009) and children with developmental language impairments (Kambanaros et al., 2013b). These include word frequency, age of acquisition, imageability, and syllable length, which were also all controlled for in the research reported here.

Within the functional architecture of the lexical processing system (Dell, 1986; Levelt, 1989; Caramazza, 1997), noun–verb word-retrieval deficits are assumed at any one of the three relatively distinct levels: during lexical-conceptual selection (as laid out above), during lemma activation (i.e., when a word's semantic and syntactic features are processed such as word class, gender, etc.), or at the lexeme level (i.e., when accessing the (morpho)phonological word forms).

There is still considerable debate in the literature—drawing, among others, from the fields of linguistics, pathology, and neuroscience—as to whether grammatical category deficits are a true breakdown of a specific grammatical category (e.g., verbs vs. nouns or the reverse) or whether dissociations can be attributed to, say, lexical, semantic, or syntactic differences between the two word classes (for a recent detailed presentation of some of the issues, see Kemmerer, 2014). Linguistic explanations for the noun–verb dissociation in language production have focused on semantic-conceptual, lexical-grammatical, and/or lexical-(morpho)phonological differences between the two word classes (for overviews, see Kambanaros, 2009b; Mätzig et al., 2009). Taking our lead from Laiacona and Caramazza (2004), we present the two central accounts that have been postulated for the noun–verb dissociation in impairment to set the stage for potential noun/verb retrieval deficits in picture naming tasks that will be reported from the LI groups participating in our studies: (i) the semantic-conceptual account and (ii) the grammatical account. The dissociation between verbs and nouns is claimed to be of fundamental importance, given that both word types essentially are universally available categories across all languages (for a review on the empirical evidence for both accounts, see Vigliocco et al., 2011).

Concerning (i), the semantic-conceptual account, and simplifying matters somewhat, verbs express states and events, that is, what happens to things, including actions, whereas nouns refer to entities such as people, animals, objects, and concepts. Or, as Hinzen and Sheehan (2014, p. 70) classify them, “nominals are ‘first phase’ denotations, which are presupposed as parts in ‘second phase’ denotations, i.e., the verbal phase.” Verbs and nouns are differentiated by contrasting sets of semantic features (e.g., verbs may be defined predominantly by functional, thematic, or action-related features and nouns more by sensory-perceptual features), suggesting that the noun–verb dissociation can arise from impairments to different domains of meaning (Marshall, 2003). In fact, verbs and nouns differ on other dimensions such as word frequency and imageability—with nouns usually higher in frequency and considered more imageable—, that is, lexical-semantic variables at large that may affect the naming process for each word type. The general lower imageability and frequency of verbs makes them more vulnerable than nouns to being impaired following brain damage (Luzzatti et al., 2002; Bird et al., 2003; Crepaldi et al., 2006). Also, age of acquisition is known to be a significant predictor of naming performance, with nouns usually acquired earlier than verbs across most languages (McDonough et al., 2011).

Alternatively, it has also been argued that prototypical nouns happen to be objects and prototypical verbs tend to be actions (for background and explanations, see Kemmerer, 2014, building mostly on the theory developed by Croft, 1991, 2001). Consequently, the selective noun–verb dissociation is due to lexical differences in referencing objects and actions, respectively, especially for confrontation picture naming where verbs/actions are represented in “static” format.

The grammatical account, (ii), points to differences between nouns and verbs in relation to argument structure and additional morphological processing associated with verbs (see Pulvermüller et al., 2012 and references within). For example, it has been claimed (Kim and Thompson, 2000) that the number of arguments a verb takes affects its retrieval even for single-word naming using pictures. In Indo-European languages, verbs are usually marked overtly for tense, aspect, mood, and number, while nouns are marked for case, gender, and number, although languages vary in which features are marked overtly.

An interesting alternative proposal that has emerged from the theoretical literature in linguistics, which might also supplement the grammatical account, comes from Kayne (2009). Essentially he argues that nouns are the only open-class items in the lexicon, and that, as a consequence, verbs should be viewed as closed-class items. There is thus a categorial distinction between nouns and verbs that goes beyond their categorical differences. This suggestion has already been picked up for agrammatic performance (Franco et al., 2012; Franco, 2014). Hinzen and Sheehan's (2014) distinction between nominal and verbal phrases as first-phase and second-phase denotations, respectively, is similar in spirit and may help determine why, with overwhelming majority, verb retrieval tends to be “harder” than the retrieval of nouns. We will return to this in the Discussion.

The participants in the present study are all speakers of Modern Greek, a stem-based language with complex verbal and nominal morphology; the children with SLI are native speakers of the Cypriot Greek variety, all adults come from Greece and speak Standard Modern Greek. All morphophonological word forms in Greek are inflected according to grammatical category; for example, from the common root *skup–*, the verb *skup–izi* “he/she sweeps” is formed, while *skup–a* “broom” yields a noun (though not all roots allow for both categories). Moreover, verbs and nouns are considered to have similar inflectional-morphological complexity (see Ralli, 2003). Information about grammatical category and syntactic features, such as person, tense, aspect, and mood for verbs or gender, number, and case for nouns, is a prominent property of Greek. For a noun/verb to be retrieved in word production, both grammatical category information and the inflectional processes that need to be applied to derive the word form must be accessed.

Let us briefly lay out the rationale for our contribution and present the three main aims. In this article we bring together the results from studies in four clinical groups involving 89 participants in total that investigate noun/verb spoken naming on a picture-based naming task (the GOAT from Kambanaros, 2003). We compared each LI group with non-impaired controls in different published studies for each impairment respectively and will not focus on the results of the non-impaired groups in this article (see Kambanaros and van Steenbrugge, 2006; Kambanaros, 2008; Kambanaros et al., 2008, 2010, 2013b, unpublished). We are primarily interested in mapping the results of the different LI groups together to help us delineate where in the lexical system lies the level of breakdown for grammatical class words. Is it in the semantic or phonological system or in the connection between the semantic and phonological output lexicons? Alternatively, is it a lemma- or lexeme-based impairment or is it difficulties with lexical access *per se* or storage deficits? Is it similar or different across groups? For the LI groups under investigation we excluded individuals with deficits at the conceptual level and those with significant articulation difficulties (e.g., dysarthria or apraxia of speech).

Ultimately, the organization and processing of nouns and verbs for the concepts representing the depicted objects and actions are part of the language faculty, the ability to express language, and the uniquely human capacity of thought. We thus expect our findings to inform future research on the relation between word class and the language of thought, even if we approach it “only” in a roundabout way. However, by exploring the suggested closed-class character of verbs, unlike nouns, some of the exiting processing accounts lose their immediate relevance to the issue (See Discussion).

The aims of the present study are three-fold:

(A1) to compare quantitative and qualitative differences for noun/verb retrieval across LI groups;
(A2) to examine noun/verb naming errors with reference to psycholinguistic models of word processing;
(A3) to shed light on the nature of the naming deficit for LI groups and determine relevant commonalities and differences.

## Methods

The demographic information of each LI group is presented in Table 2.

**Table 2:** **Demographic characteristics of reported LI groups**.

**LI Group**	**Mean age (range)**	**Gender**	**Years of education (range)**	**Intelligence level (range)**
Broca's aphasia	62.4 (30–81)	2 females 5 males	6.0 (4–8)	N/A
Anomic aphasia (bilingual)	70.5 (60–84)	4 females 8 males	6.0 (4–8)	N/A
Anomic aphasia (monolingual)	60.4 (57–68)	3 females 2 males	6.0 (4–8)	N/A
Schizophrenia	39.0 (25–62)	14 males 6 females	10.8 (6–16)	98.25 (88–105)
Relapsing remitting multiple sclerosis	40.8 (17–56)	24 females 7 males	12.25 (9–18)	101.5 (80–110)
Children with specific language impairment	6.9 (5.5–9.9)	4 females 10 males	Primary school (grades 1–4)	Non–verbal IQ > 80

*LI, language-impaired; N/A, not applicable*.

Please note that the difference in education between the aphasic patients and the other groups stems from the generational gap observed in and typical for Greek adults of the time. In the Greece of 50 years ago, in which these participants grew up, it was not uncommon to receive only rudimentary formal schooling. The anomic aphasics were residents of Australia at the time of testing, and had been so for many decades; they were the traditional low-educated labor migrants. The Broca's aphasics were tested later and matched in education to this original clinical population.

### Individuals with Aphasia (N = 24)

The participating individuals with aphasia had suffered a single, relatively localized lesion in the left hemisphere with no other neurological involvement. All were chronic aphasics and met the following criteria: no previous history of infarct, neurologically and physically stable (over 6 months post onset), no history of active or significant alcohol and/or drug abuse, no history of active psychiatric illness or other brain disorder (e.g., Parkinson's disease, Huntington's disease, Korsakoff's syndrome, Alzheimer's disease and other presentations of dementia, senility, and mental retardation), corrected-to-normal auditory and visual acuity for age. All participants were right-handed by self-report and native speakers of Greek. The individuals with Broca's aphasia all showed a right hemiplegia, but those with anomic aphasia had milder right-side involvement of the hand and leg. The diagnosis of aphasia type was based on the results of the Greek version of the Boston Diagnostic Aphasia Examination (BDAE; Tsolaki, 1997), whose severity rating scale ranges from 0 to 5, with 0 = no usable speech, 3 = mild aphasia, and 5 = minimal speech handicaps. The participants with Broca's aphasia presented with non-fluent speech and a mild–moderate to severe aphasia (BDAE severity rating 3–5). The participants with anomic aphasia presented with fluent speech and moderate naming deficits (BDAE severity rating of 4). For the Greek–English bilingual anomic aphasic group, English was also evaluated using the original BDAE (Goodglass and Kaplan, 1983). The results showed that aphasia was apparent in both languages, with language difficulties more evident in L2 (English); severity ratings ranged between 3 and 4. See Kambanaros and van Steenbrugge (2006), Kambanaros (2008), and Kambanaros et al. (2008) for detailed assessment results on the BDAE.

### Individuals with Schizophrenia (N = 20)

This group were out-patients from the Mental Health Centre in Patras, Greece, who met established criteria for schizophrenia from the Diagnostic and Statistical Manual, 4th edition (American Psychiatric Association, 2010). They were all non-compensated volunteers who had been referred by the consultant psychiatrist. All patients were clinically evaluated for psychiatric status according to DSM-IV-TR criteria (using SCID Axis I and SCID Axis II) and by a specialist neurologist to exclude neurological disorders. Schizophrenia type varied within the group: 10 individuals were reported as suffering from paranoid schizophrenia, six individuals with undifferentiated schizophrenia, and two individuals with catatonic and residual schizophrenia, respectively. In addition, 17 of the individuals with schizophrenia were on atypical antipsychotic medication, while the remaining three were on typical antipsychotic and mood stabilizer medication. Exclusion criteria from the naming study included: organic CNS pathology-neurological disorders, HIV/HCV infection, major psychopathology spectrum disorders (excluding schizophrenia), head trauma resulting in loss of consciousness for longer than 5 min, dementia, mental retardation, and current therapy with medications or medical conditions known to affect cognition, illicit substance dependencies including alcohol for the past 6 months prior to inclusion in the maintenance therapy, and non-native speakers of the Greek language. All had adequate hearing and vision for test purposes and had provided informed consent to participate in the study, and permission to conduct the study was obtained by the local ethics committee. Participants with schizophrenia were also administered a brief battery of neuropsychological tests in order to assess verbal fluency, verbal learning/memory, psychomotor speed, attention, executive functioning, and mood (severity of depression). The reader is referred to Kambanaros et al. (2010) for detailed demographic and clinical characteristics of the participants.

### Individuals with RRMS (N = 31)

This group consisted of patients with relapsing remitting multiple sclerosis (RRMS), diagnosed according to the McDonald criteria (McDonald et al., 2001), from the Neuropsychology Unit, Department of Neurology, University of Patras Medical School. Patients with acute relapse during the past 3 months before the study, patients on corticosteroids or on other medications that could interfere with cognition, or patients with learning disabilities, visual deficits, limb paralysis, major psychiatric illness, or other neurological diseases were not included in the study. All participants provided written consent, which was approved by the Ethics Committee of the University of Patras. Participants with RRMS were also administered a brief battery of neuropsychological tests in order to assess verbal fluency, verbal learning/memory, psychomotor speed, attention, executive functioning, and mood (severity of depression). The reader is referred to Kambanaros et al. (unpublished, but a pre-publication copy can be requested from the authors) for detailed demographic (age, education, gender distribution, intelligence level) and clinical characteristics of the RRMS patients (Expanded Disability Status Scale, disease duration, Beck Depression Inventory–Fast Screen).

### Children with SLI (N = 14)

Children were diagnosed with SLI prior to the noun/verb naming study using a language battery of norm-referenced tests (see Kambanaros et al., 2014 for assessment specifics and results): The language assessment battery included measures of (a) receptive vocabulary (Greek version of the Peabody Picture Vocabulary Test; Simos et al., 2011), (b) expressive vocabulary (Diagnostic Language IQ Test/DVIQ; Stavrakaki and Tsimpli, 2000), (c) comprehension and production of morphosyntax (DVIQ), (d) metalinguistic concepts (DVIQ), (e) sentence repetition (DVIQ), (f) articulation and phonological processing (Phonological and Phonetic Test; Panhellenic Association of Logopedists, 1995), (g) word definitions (subtest of the Athina Test; Paraskevopoulos et al., 1999), (h) word finding (Greek version of the Renfrew Word Finding Vocabulary Test; Vogindroukas et al., 2009), and (i) phoneme discrimination (subtest of the Athina Test; Paraskevopoulos et al., 1999). All Greek tests were adapted into Cypriot Greek where possible or relevant (lexical and/or phonological alternatives), which did not have any impact on the single-word verb/noun picture naming tasks (Theodorou, 2013; see also Kambanaros and Grohmann, 2013). The language difficulties encountered by the children were predominantly in expressive language in the domains of (morpho)syntax and the lexicon. Hearing and vision were adequate for test purposes and the children with SLI exhibited normal performance on a screening measure of non-verbal intelligence (Raven's Colored Progressive Matrices; Raven et al., 2000). Children also showed normal articulation, had no gross motor difficulties, and came from medium to high socio-economic status families. They were recruited from speech and language therapists servicing public primary schools and therapists from private practices. All children were in mainstream education and in the school grade corresponding to their chronological age. Twelve of the children were receiving speech and language therapy at the time of the study, and three attended special education classrooms for part of the day. Participant selection criteria included a (Cypriot) Greek-speaking family background and no history of neurological, emotional, or behavioral problems.

### Materials

The Greek Object and Action Test (GOAT, Kambanaros, 2003) was administered to assess retrieval of nouns and verbs: The adapted version of the GOAT used to test the children with SLI had 35 nouns (instead of 42) as nouns with a mean age of acquisition greater than 6 years were removed (for the Cypriot Object and Action Test, see Kambanaros et al., 2013b). It contains 84 colored photographs, 10 × 14 cm in size representing 42 actions (verbs) and 42 objects (nouns). The GOAT was piloted on a group of twenty non-brain-injured, monolingual Greek speakers aged between 55 and 75 years (Kambanaros, 2003). Only items named with 80% accuracy or more were included in the test. Objects are concrete inanimate nouns and include manipulated instruments used for activities of daily living such as garage tools (e.g., hammer), garden equipment (e.g., rake), kitchen utensils (e.g., grater), and items from household (e.g., broom), office (e.g., pen), or personal use (e.g., comb). Verbs are monotransitive, though frequently allow their object dropped, and actions are restricted to past stereotypical roles, that is, a woman is shown performing household activities (e.g., mopping) and a man is performing more manly duties (e.g., hammering). These stereotypical roles depicted in the photographs are deemed to be appropriate for the ages and cultural groups tested. All target nouns in object naming were also items in the noun comprehension task. All target verbs in action naming were also targets in verb comprehension task.

Verb and noun word frequencies were calculated based on the printed word frequency count for Standard Greek (Hatzigeorgiou et al., 2000). A Mann-Whitney test revealed no significant difference between nouns and verbs (*z* = −0.154, *p* = 0.878). In addition, there was no significant difference in syllable length between nouns and verbs (*z* = −0.610, *p* = 0.542). Nouns and verbs were measured also for age of acquisition (AoA, estimated age ratings were based on first contact with the given noun/verb in either verbal or written form using a seven-point scale, with 1 representing 0–2 years of age, 2 being 3–4 years of age, up to 7 for 13 years of age and older), imageability (ratings were performed on an eight-point scale, with 0 = impossible, 1 = least imageable, up to 7 = most imageable), and picture complexity (ratings were performed on a seven-point scale related to the ease with which the noun/verb picture was recognized, from 1 = least ease to 7 = most ease). A Mann-Whitney test revealed that the nouns and verbs were not significantly different on AoA (*z* = −1.168, *p* = 0.243), but there was a significant difference for word imageability (*z* = −2.978, *p* = 0.003) and picture complexity (*z* = −2.331, *p* = 0.20), with higher ratings for nouns compared to verbs, revealing nouns as more imageable and visually less complex than verbs upon picture identification. Such differences between verbs and nouns for imageability and picture complexity—by virtue of depicting actions in a static fashion—are common phenomena reported in the literature across languages which investigate verbs and nouns with pictured stimuli (for a review, see Crepaldi et al., 2011).

### Procedure

The order of the task—noun or verb testing in the GOAT—was counter-balanced across the participants. Each participant was tested individually by a certified speech and language therapist (the first author, a certified bilingual speech pathologist, administered the GOAT on all LI populations). The same set of 84 items of the GOAT (42 verbs and 42 nouns, respectively 39 verbs and 35 nouns for the children with SLI) was used for both the comprehension and the word production tasks. Production and comprehension tasks were tested at least 1 week apart, with comprehension tasks preceding naming tasks. The noun and verb tasks per subtest were counterbalanced across all participants.

For comprehension, participants were asked to point to the correct photograph from a set comprising the target noun or verb, and the two semantic distractors for each target noun/verb. Each participant was asked to point to the picture of the noun or verb matching the spoken word heard. Two examples were provided before testing. If participants failed to point to the correct picture, they were corrected. Participants who pointed to more than one photograph were told that only one picture was correct. The instructions were repeated for participants who did not point to any pictures. No time limits were placed, hence no reaction times measured, and self-correction was allowed. Only once was the target word repeated upon subject request. If further repetitions of the same word were required, the answer was scored as incorrect.

For word production, participants were asked to name the noun or verb represented by the object or action depicted in the photograph, respectively, in a single word. The stimulus question was short and of equal length for both the noun and verb naming subtests (five syllables): *Ti ine afto?* “What's this?” for objects and *Ti kani aftos/afti?* “What's he/she doing?” for actions. In the responses, nouns were supposed to be provided marked for nominative (which all participants did, though other case-markings would have been accepted too). Since Greek lacks a non-finite citation form (infinitive or gerund), verbs were required in the third person singular present tense (which all participants did, though other inflections would have been accepted too). Two examples were provided before testing. The stimulus was repeated once for participants who did not respond. If no response was given, the item was scored as incorrect. Again, no time limits were placed, hence no reaction times measured, and self-correction was allowed.

## Results

**Figure 1:**
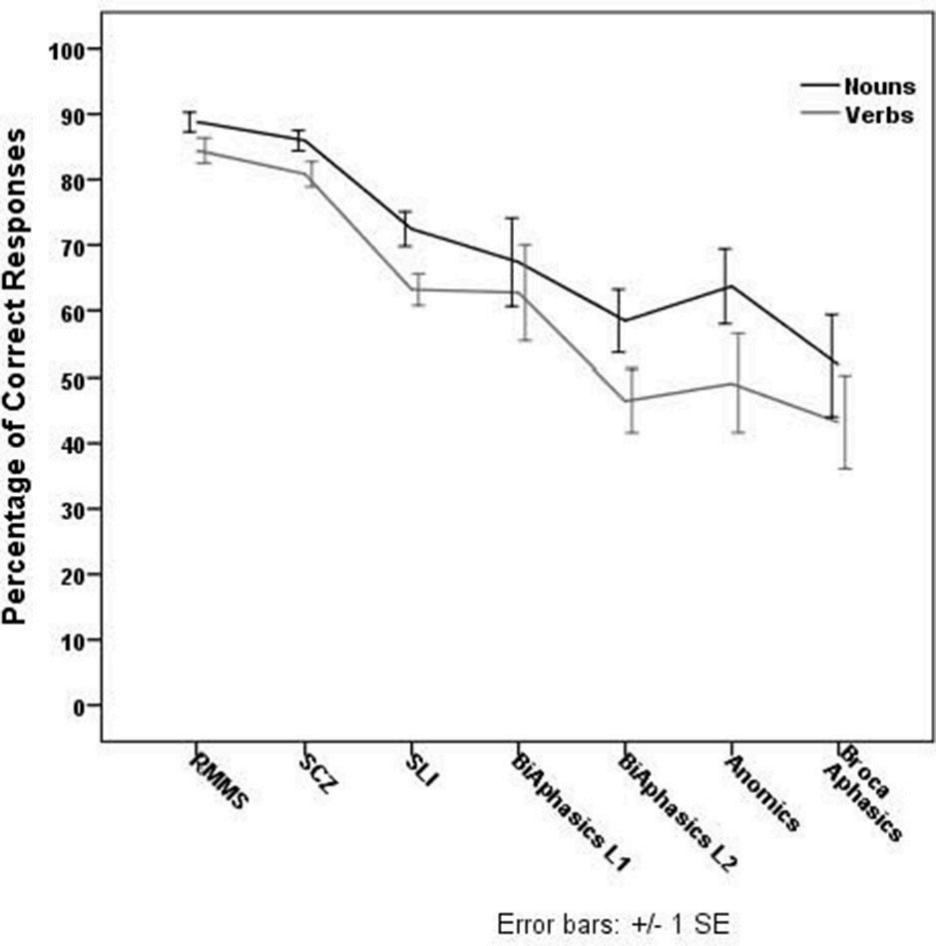
**Percentage of correct responses for nouns and verbs retrieved by each LI group on the GOAT**.

Concerning accuracy, correct responses for the comprehension task were photographs that matched the target verb or noun picture spoken by the examiner. Given that comprehension of grammatical class words was close to or at ceiling for most groups, the results will not be discussed further; the LI groups showed little impairment on the comprehension tasks of the goat (for each published group study, see Kambanaros and van Steenbrugge, 2006; Kambanaros, 2008; Kambanaros et al., 2010, 2013b). The percentage of correct responses for nouns and verbs retrieved by each LI group on the GOAT is reported in Figure 1. A Wilcoxon Signed Rank Test (Wilcoxon, 1945), which reports significance at *p* = 0.05 for paired samples was performed to see whether there was a difference between noun and verb naming accuracies within each LI group.

### Aphasia

There was a significant difference between verb and noun naming accuracies for participants with Broca's aphasia (*z* = −2.37, *p* = 0.018), anomic aphasia (*z* = 2.023, *p* = 0.043), and bilingual anomic aphasia (L1: *z* = −2.51, *p* = 0.012; L2: *z* = −2.82, *p* = 0.005). Verbs were significantly more difficult to retrieve than nouns in picture naming for all three groups with aphasia.

### Schizophrenia

There was a significant difference between verb and noun naming accuracies (*z* = −2.98, *p* = 0.003), with verbs significantly more difficult to retrieve than nouns in picture naming.

### Relapsing remitting multiple sclerosis

There was a significant difference between verb and noun naming accuracies (*z* = −2.99, *p* = 0.003), with verbs significantly more difficult to retrieve than nouns in picture naming.

### Specific language impairment

There was a significant difference between verb and noun naming accuracies (*z* = −2.42, *p* = 0.016), with verbs significantly more difficult to retrieve than nouns in picture naming.

Overall, verbs were significantly more difficult to retrieve compared to nouns for all LI groups (Pearson chi-square test: χ^2^ = 1.0819, df = 5, *p* = 0.956). Multiple linear regression models were run to predict whether the lexical-semantic variables (word frequency, syllable length, age of acquisition, imageability) and picture complexity associated with the verb and noun stimuli influenced naming accuracy for the LI groups. The results are provided in Table 3.

**Table 3:** **Statistical analysis of role of variables for verb and noun naming accuracy**.

	**BA**		**SCZ**		**RRMS**		**SLI (ch.)**	
	**Verbs**	**Nouns**	**Verbs**	**Nouns**	**Verbs**	**Nouns**	**Verbs**	**Nouns**
Word frequency	*t* = 0.51 *p* = 0.82	*t* = 0.3 *p* = 0.76	*t* = 0.52 *p* = 0.60	*t* = −0.7 *p* = 0.9	*t* = 1.64 *p* = 0.00^*^	*t* = 0.19 *p* = 0.6	*t* = −0.01 *p* = 0.93	*t* = 0.16 *p* = 0.25
Age of acquisition	*t* = −0.3 *p* = 0.54	*t* = 0.62 *p* = 0.87	*t* = 2.6 *p* = 0.00^*^	*t* = 0.13 *p* = 0.9	*t* = −3.44 *p* = 0.00^*^	*t* = 0.15 *p* = 0.56	*t* = 0.52 *p* < 0.01^*^	*t* = 0.57 *p* < 0.01^*^
Imageability	*t* = −0.23 *p* = 0.98	*t* = −0.62 *p* = 0.54	*t* = 0.13 *p* = 0.89	*t* = −0.53 *p* = 0.6	*t* = 0.24 *p* = 0.00^*^	*t* = −0.060 *p* = 0.69	*t* = 0.10 *p* = 0.53	*t* = 0.09 *p* = 0.51
Picture complexity	*t* = −0.62 *p* = 0.61	*t* = −1.0 *p* = 0.31	*t* = −3.9 *p* = 0.00^*^	*t* = −4.3 *p* = 0.00^*^	*t* = −3.41 *p* = 0.00^*^	*t* = −4.06 *p* = 0.00^*^	*t* = 0.13 *p* = 0.39	*t* = 0.18 *p* = 0.21

BA, Broca's aphasia; SCZ, schizophrenia; RRMS, relapsing remitting multiple sclerosis; SLI, specific language impairment; ch., children;

* *significant at the 0.01 level*.

In addition, a univariate ANOVA was performed with Accuracy Score (percentage of correct responses) as a dependent variable, and Condition and Word Class (verbs vs. nouns) as fixed factors. This resulted in significant main effects of Condition [*F*_(6, 204)_ = 35.1, *p* < 0.01, η^2^ = 1] and Word Class [*F*_(1, 204)_ = 13.53, *p* < 0.01, η^2^ = 0.96)], but no interaction between Condition and Word Class [*F*_(6, 204)_ = 0.48, *p* = 0.85].

Bonferroni *post-hoc* tests showed that the RMMS and the SCZ groups performed better overall than all the other groups (*p* < 0.01), but similarly to each other (*p* = 1), whereas the groups of bilingual aphasics, anomic aphasics, and Broca's aphasics performed similarly to each other (*p*s > 0.5), but worse than all the other groups (*p* < 0.01).

### Aphasia

For participants with Broca's aphasia, neither any lexical-semantic variable nor picture complexity affected naming performance for verbs and nouns. For participants with anomic aphasia, none of the psycholinguistic variables predicted performance for verbs; however, for nouns, age of acquisition, picture complexity, and imageability all predicted performance (*p*s < 0.01).

### Schizophrenia

Picture complexity was a significant predictor of noun and verb naming accuracy. Age of acquisition also significantly predicted accuracy for verbs—but not for nouns. None of the other variables significantly predicted accuracy.

### Relapsing remitting multiple sclerosis

Picture complexity and age of acquisition were significant predictors of naming accuracy for both verbs and nouns. In contrast, word frequency was a significant predictor for successful verb but not noun retrieval. None of the other variables significantly predicted accuracy.

### Specific language impairment

Only the mean age of acquisition of a word was significant in predicting naming accuracy for both noun and verbs. None of the other variables significantly predicted accuracy.

The comparative findings regarding the effect of lexical-semantic variables and picture complexity across LI groups are depicted in Table 4.

**Table 4:** **Effect of variables for language-impaired group performance on verb and noun naming**.

	**BA**		**SCZ**		**RRMS**		**SLI (ch.)**	
	**Verbs**	**Nouns**	**Verbs**	**Nouns**	**Verbs**	**Nouns**	**Verbs**	**Nouns**
Word frequency	x	x	x	x	✓	x	x	x
Age of acquisition	x	x	✓	x	✓	✓	✓	✓
Imageability	x	x	x	x	x	x	x	x
Picture complexity	x	x	✓	✓	✓	✓	x	x

*BA, Broca's aphasia; SCZ, schizophrenia; RRMS, relapsing remitting multiple sclerosis; SLI, specific language impairment; ch., children; ✓, significant in predicting accuracy influence; x, no influence*.

Let us now address the qualitative error analysis. The naming errors produced on the GOAT by LI groups were classified into:

- semantic substitutions (e.g., the output “broom” for the targeted entry “mop,” “threading” for “sewing”);
- semantic descriptions/circumlocutions (e.g., “use with pencils” for “sharpener,” “making a house” for “building”);
- phonological errors, including words that shared the same onset and number of syllables with the target word (e.g., the output /ti'ri/, incidentally meaning ‘cheese, for the entry /sfi’ri/ “hammer”);
- grammatical word class substitutions, either noun–verb (e.g., instead of the noun “needle,” the verb “sewing” was produced) or verb–noun (e.g., instead of the verb “sweeping,” the noun “broom” was produced);
- word-form errors (mainly “don't know,” no responses, gestures, or visual misinterpretations of the target);
- unrelated errors (unclassifiable errors).

The number and type of errors produced by the different LI groups on the verb and noun naming tasks are reported in Table 5.

**Table 5:** **Mean percentage of the different error types (standard deviations in parentheses where available) committed by language-impaired groups on verb and noun naming**.

**LI Group**	**BA**	**Biling AA (L1 & L2, combined)**	**Monoling AA**	**SCZ**	**RRMS**	**SLI (children)**
**ERROR TYPES: VERBS**						
Semantic circumloc.	1.7 (2.3)	15.6	24.8	9.2 (9.1)	0.5 (1.2)	17.0 (8.3)
Semantic errors	11.2 (8.7)	7.8	12.9	7.4 (5.1)	10.2 (5.7)	11.2 (7.8)
Phonological errors	2.0 (2.5)	5.5	5.6	0	0	0
Word-form errors	26.2 (20.1)	1.9	4.9	0	2.5 (5.5)	7.9 (7.4)
Gramm. class errors	0.6 (1.2)	0.5	2.7	1.1 (3.2)	0	0.4 (0.9)
Unrelated errors	7.8 (11.8)	0	0	0.1 (0.5)	0	0.2 (0.7)
Code-switching	–	12.6	–	–	–	–
**ERROR TYPES: NOUNS**						
Semantic circumloc.	0	3.8	11.5	4.2 (3.7)	0	4.9 (10.2)
Semantic errors	4.4 (6.1)	7.4	13.4	6.5 (4.6)	0	9.8 (7.0)
Phonological errors	1.4 (1.9)	9.5	5.5	0	0	0
Word-form errors	31.0 (21.2)	2.4	4.4	0	4.1 (7.0)	10.2 (7.9)
Gramm. class errors	1.7 (3.6)	0.5	1.5	0	0	0.6 (1.2)
Unrelated errors	8.2 (13.2)	0	0	0.8 (1.4)	0	1.0 (2.4)
Code-switching	–	13.0	–	–	–	–

*LI, language-impaired; BA, Broca's aphasia; Biling, Bilingual; AA, anomic aphasia; Monoling, Monolingual; SCZ, schizophrenia; RRMS, relapsing remitting multiple sclerosis; SLI, specific language impairment; circumloc., circumlocutions; Gramm., Grammatical*.

### Aphasia

Most errors combined across the three aphasic groups during noun and verb spoken naming were semantic substitutions (paraphasias and circumlocutions), followed by word-form errors for both categories, with the highest number of word-form errors committed by individuals with Broca's aphasia, and equally for verbs and nouns. There were relatively few grammatical class substitutions, and some phonological paraphasias, mainly for nouns and only by the bilingual anomic group. For this group, code-switching occurred mainly from the second to the first language, with no significant difference in the number of code-switched responses between verbs and nouns.

### Schizophrenia

While individuals made in total many more errors in response to verbs than to nouns, errors in both word classes were predominantly of a semantic nature (paraphasias and circumlocutions), and no word form errors were evident for either category. Verbs and nouns showed a reverse effect with regards to semantic error type: Verb naming generated greater semantic circumlocution errors followed by semantic paraphasias, whereas for noun naming more semantic paraphasias were produced than semantic circumlocutions. Also, more unrelated responses were produced for nouns compared to verbs, while grammatical word class substitutions (albeit very few) were evident for verbs only.

### Relapsing remitting multiple sclerosis

For this group, semantic paraphasias were the most common error for verbs, whereas word-form errors predominated for nouns. Also, for nouns there was no evidence of semantic or grammatical word class errors. Similarly, for verbs there were no grammatical word class errors.

### Specific language impairment

Children with SLI produced significantly more semantic errors for verbs, predominantly semantic circumlocutions compared to nouns, followed by semantic paraphasias, whereas for nouns the reverse order was observed. Also, the number of word-form and grammatical word class errors—which were both very few—were not significantly different between nouns and verbs.

## Discussion

We reported on the noun–verb dissociation in spoken word production for Greek, a morphologically complex language, in adults with acquired language impairments and a group of children with SLI, a developmental language disorder. We administered a battery of noun and verb pictures (the GOAT), matched on frequency, age of acquisition, syllable length, imageability, and picture complexity. While much research has been conducted with aphasic populations on the noun–verb dissociation in spoken naming, studying different language impaired groups beyond aphasia provides valuable information on how different types of brain/and or genetic involvement impact on word-retrieval abilities. Overall, the grammatical class effect in aphasia has been attributed to either grammatical, or lexical, or semantic (viz. imageability) differences between verb and noun representations. We will proceed with a *narrow discussion* that addresses the three stated aims one by one before turning to a *broad discussion* in which we will sketch the beginnings of an alternative route of explanation.

### Narrow discussion

In relation to our first aim (A1), to compare quantitative and qualitative differences for noun/verb retrieval across LI groups based on results from the available behavioral data, we can observe that verbs are more vulnerable to retrieval breakdown compared to nouns in acquired and developmental language disorders. More specifically, the analysis concentrates on the findings of a disproportionate impairment in naming verbs compared to nouns in light of minimal word comprehension difficulties of both word classes. The current results appear to support a complex and distributed network for noun and verb processing (Crepaldi et al., 2013). They also lend little support to established neuroanatomical explanations of noun–verb production deficits in spoken naming.

With regards to number and patterns of naming errors for verbs and nouns across LI groups, errors of a semantic nature predominated, with a higher number of circumlocutions produced more often in response to verbs compared to nouns, whereas semantic paraphasias were produced more often for nouns—but not by all LI groups. Specifically, for four out of the six LI groups, circumlocutions were the predominant naming error for verbs: adult participants with monolingual anomic aphasia, with bilingual anomic aphasia, and with schizophrenia as well as children with SLI. In contrast, for nouns this error type was small (< 5%) for three groups (bilingual anomic aphasia, schizophrenia, and SLI), and it was not at all committed by two groups (Broca's aphasia and RRMS); these two groups instead produced a small number of semantic paraphasias for verbs. Furthermore, word form errors for verbs were the highest for the Broca's aphasic group for which it constituted their largest verb error type. As for the remaining groups, word-form errors were few (between 2 and 8%) and for the SCZ group non-existent. What is more, grammatical word class errors for verbs were few across groups (< 3%), but the RRMS produced no such error. In turn for nouns, both the RRMS and SCZ groups revealed no grammatical word class errors, while all remaining groups had very few such errors (< 2%). For the Broca's aphasia group, word form errors were again significantly high, this time for nouns (above 25%), making it their largest noun error type, whereas the remaining groups showed fewer word form errors (< 10%) and the SCZ group made no such errors at all for nouns. Semantic errors for nouns (paraphasias and circumlocutions) predominated for the monolingual anomic aphasic and SLI groups, whereas the Broca's aphasic and RRMS groups revealed few or no semantic errors of either type for nouns. Semantic paraphasias for nouns were few for the Broca's aphasic, the bilingual anomic, and the SCZ groups (< 8%). Semantic circumlocutions were even fewer for the SCZ and the bilingual anomic groups (< 5%). Taken as a whole, the Broca's aphasic group showed the largest naming impairment and as such produced the highest number of errors for verbs and nouns across groups, followed next by the anomic aphasic groups (monolingual first and then bilingual), and then by the children with SLI. The smallest noun/verb naming impairment was evidenced in the RRMS group, followed by the SCZ group.

In keeping with our second aim (A2), to examine noun/verb naming errors with reference to psycholinguistic models of word processing, the seminal model developed by Levelt (1989), later expanded by Levelt et al. (1999), will serve as the point of reference. The model posits that lexical selection of a target word is a competitive process influenced by the activation of competitors in the mental lexicon sharing semantic and/or phonological features with the target response. Selective impairments of verbs or nouns may arise at a number of relatively distinct levels or components postulated within this serial model of word processing. They may surface when accessing/retrieving the conceptual or semantic information for the target word, when retrieving the lemma (grammatical) information, when accessing the morphological and/or syntactic components of grammar, or when accessing/retrieving the phonological representation of the target word within the phonological output lexicon. Furthermore, different lexical-semantic or psycholinguistic variables of the word properties can affect retrieval during each stage of the naming process.

The following variables were taken into account using logistic regression procedures when analyzing verb and noun naming accuracies within and across the LI groups: word frequency, age of acquisition, imageability, and picture complexity (see Table 3). Word frequency is assumed to operate at the level of the phonological output lexicon, hence frequent (high-frequency) words are retrieved more readily than less frequent (low-frequency) words. Similarly, AoA, which is closely associated with word frequency, holds that words acquired early in life are easier to retrieve than later acquired words, where early acquired words also tend to be of higher frequency (Morrison et al., 1997). Yet, frequency-independent AoA effects are posited at the lemma level for picture naming (Bates et al., 2001). Imageability, on the other hand, is said to be a feature operating at the level of lexical semantics, with highly imageable (concrete) words easier retrieved than (abstract) words with low imageability (see Howard and Gatehouse, 2006). Picture complexity arguably has an effect at the semantic-conceptual processing stage, where verbs are represented by (static) action pictures and nouns by (concrete) object pictures.

The results revealed that *word frequency* predicted verb naming accuracy only, and only for one LI group, the participants with RRMS, who found verbs with a higher frequency easier to retrieve than verbs with a low frequency. No other LI group showed an effect of word frequency on noun/verb naming. This suggests that the verb deficit was at a post-semantic level for the RRMS group. Verbs with high frequency were associated with better production in previous studies involving individuals with Broca's (Luzzatti et al., 2002; Park et al., 2013) and anomic aphasia (Luzzatti et al., 2002), but this did not surface in our research.

Also, *age of acquisition* influenced verb naming accuracy in the RRMS group as well as in the SCZ and SLI groups, with earlier acquired verbs easier retrieved than later acquired verbs. For the SLI group, noun retrieval was also influenced by age of acquisition, with earlier acquired nouns more easily retrieved than later acquired nouns. There is evidence that lemmas of early acquired words are more potent competitors to other lemmas than those of later acquired words (for a detailed description, see Belke et al., 2005, p. B52). For the SLI group this allowed noun/verb lemmas of early acquired words to be activated more intensively for successful word production. Similarly, high activation of verb lemmas of early acquired words facilitated naming for the SCZ and RRMS groups. It is also possible that the AoA and frequency effect are intercorrelated for the RRMS group and relate to a general lexical deficit for verbs. No aphasic group showed an effect of age of acquisition on noun/verb naming accuracies.

Furthermore, the RRMS group was the only LI group to show an effect of *imageability* on naming accuracy—again, for verbs only, with verbs rated highly imageable more easily retrieved than verbs rated low in imageability. This finding for verb naming has also been reported for some individuals with Broca's (Luzzatti et al., 2002; Park et al., 2013) and anomic aphasia (Luzzatti et al., 2002) but was not apparent in our aphasic groups. Although imageability effects have traditionally been taken to indicate a semantic impairment, our group of RRMS individuals achieved ceiling effects on noun/verb comprehension tasks, not reflecting (superficially at least) a semantic deficit.

Finally, *picture complexity* influenced both noun and verb naming accuracies for two LI groups—participants with RRMS and those with SCZ—where words with a high complexity rating for their picture were more difficult to retrieve. That is, more picture-complex words provoked the selection of non-target responses as opposed to words with lower picture complexity ratings. This finding reinforces previous claims that an additional methodological drawback in studying verb and noun differences using pictures is that concrete action and object words cannot be perfectly matched on visual picture complexity (and imageability), variables known to be important for at least some brain injured patients (Druks et al., 2006: 337). The reader is referred to Table 4 for similarities and differences across LI groups on the lexical-semantic and picture complexity measures.

Bringing together the findings across LI groups, we will pitch them against Levelt's model (Levelt, 1989; Levelt et al., 1999), which is divided into two explanations within the semantic lexicon (lemma level) vs. the phonological lexicon (lexeme level). In light of a disconnection between the semantic and the phonological lexicon, it should be noted that the noun–verb dissociation might also be couched within the Independent Network Model, according to which verbs and nouns are stored independently (Kehayia, 1990; Caramazza and Miozzo, 1997), that is, two separate storage mechanisms for lexemes according to their grammatical category. As far as we can see at this point, however, the results do not (dis)favor one model over the other. For this reason, yet with the provisos addressed right below, we will subsequently develop the beginnings of a very different route of research toward an explanation in the Broad Discussion.

### Lemma-level explanations

Since the LI groups had little difficulty comprehending single verbs and nouns, it is unlikely that the noun–verb dissociation resulted from a deficit at the semantic-conceptual store before lexical retrieval processes were initiated. Instead, given the high number of semantic errors for verbs, it is possible that the specific verb impairment across LI groups was at the level of the lemma, since verb lemmas convey greater grammatical complexity than noun lemmas (e.g., thematic roles, argument structure, and transitivity). However, there is at least one major argument challenging the suggestion of a breakdown at the lemma-level: There were very few verb–noun substitutions, hence lemma information was generally intact for both categories. However, for the RRMS group, it is possible that the verb effect was imageability-dependent, a finding that has been reported in the literature for certain individuals with aphasia (both fluent and non-fluent) on verb picture-naming tasks (Luzzatti et al., 2002).

In addition, given that verbs and nouns in Greek are of similar morphological complexity, it is hard to argue that the greater difficulty naming verbs was due to a selective impairment of some explicit morphological operations for verbs, even though half the verbs included in the study had a slightly more complex morphological structure (root + affix + affix) than the nouns and remaining verbs, respectively (root + affix). Conversely, the absence of suffixation errors makes breakdown at the level of inflectional morphology highly unlikely. Given the above interpretations, a common breakdown at the level of the lemma is difficult to reconcile with our findings across LI groups.

### Lexeme-level explanations

LI participants hardly made any phonological errors, thus ruling out a specific phonological processing deficit (for verbs) or a deficit in the phonological output buffer. In fact, because comprehension of pictures was good, and since semantic and/or word form errors were the prominent error types, the findings lead us to suggest that the locus of the naming impairment across LI groups is attributed to a deficit in the connection between the semantic and phonological output lexicons or in other words at the interface of the lexeme and lemma levels. This explanation, that LI groups showed a breakdown in mapping between intact semantic and phonological representations, calls for caution because (i) we rely on behavioral data from one task only (picture naming) for word comprehension and production, whereas (non-word) reading and repetition abilities may have provided additional stronger evidence in support of our claim; (ii) we cannot completely rule out a semantic impairment that was sufficient to impact on word retrieval which was not detected by our conventional test of comprehension; (iii) we report on group and not individual performances, and variation is always large in pathology; and (iv) we did not investigate the effects of correct cues and miscues on word retrieval which may have allowed us to tease apart individuals who were impaired at the semantic and/or phonological levels (on all points raised, see Howard and Gatehouse, 2006; Friedmann et al., 2013).

Our third aim (A3) was to shed light on the nature of the naming deficit for LI groups, in particular to determine relevant commonalities and differences, with regards to (a) the grammatical class distinction in spoken naming, (b) number and type of errors for nouns and verbs, and (c) effects of lexical-semantic variables and picture complexity on noun/verb naming accuracies. Tentative explanations are provided below based on the individual LI group findings.

We first address *commonalities and differences* found in our study. Beginning with the noun–verb dissociation effect in spoken naming, all groups showed relatively spared comprehension of grammatical word class and more difficulties naming single verbs compared to single nouns on picture-based tasks. For the aphasic groups, this goes against what was typically described in the past, namely that individuals with Broca's aphasia show better naming performance on nouns than on verbs and individuals with anomic aphasia are better on verbs compared to nouns on naming tasks. Nevertheless, the results from our aphasic groups are consistent with the increasing body of research revealing verb naming deficits in both Broca's and anomic aphasic individuals (See Aims and Background Section for references). For the remaining LI groups, our results are new and as such we have no cross-linguistic evidence to corroborate our findings—with the exception of the SLI group. For our Cypriot Greek-speaking children with SLI, verb naming on picture-based naming tasks was significantly worse compared to naming nouns, as has also been reported for English-speaking children with SLI (Sheng and McGregor, 2010).

In view of the *magnitude of the naming impairment* across LI groups, by arbitrarily setting a cut-off point of 25% and above of errors as revealing a moderate-to-severe naming impairment, three LI groups fell in this range: all aphasic groups (Broca's and anomic aphasics) and children with SLI. The finding that children with SLI, a developmental language disorder, showed a comparable lexical deficit to acquired aphasia groups is interesting and may yield new insights into the nature of word breakdown from developmental and acquired disorders, with the ultimate aim of informing intervention for both groups (for first attempts, see Kambanaros and van Steenbrugge, 2013; Bishop et al., 2014). In contrast, the SCZ and the RRMS groups presented with a mild naming impairment for nouns and verbs. It is worth noting that lexical retrieval deficits are not traditionally assumed to be a significant language symptom of multiple sclerosis or schizophrenic profiles and have thus not received much attention by researchers (see Barwood and Murdoch, 2013). However, it is possible that poor naming of nouns and/or verbs may be a marker of incipient cognitive decline for both multiple sclerosis or schizophrenia, with the effect larger for verbs compared to nouns, and that common cognitive-linguistic testing is not sensitive or specific enough to capture this linguistic phenomenon.

Taking into consideration the error patterns, a further commonality shared by all LI groups was intact grammatical word class information *per se*, as evidenced by the negligible number of grammatical word class errors (where the noun “broom” was produced once instead of the verb *sweeping*), and intact phonological processes, given the very minor phonological errors made across LI groups. Combining the two can be taken as additional evidence that the processing models available in the literature, be it the specific model we elaborated on (Levelt, 1989; Levelt et al., 1999) or some alternative (e.g., Kehayia, 1990; Caramazza and Miozzo, 1997), are not sufficient to capture both the differences but also the commonalities found in the picture confrontation naming task administered to the five groups of adults and the one group of children with language impairments. Moreover, based on the striking finding of differential error patterns in response to naming verbs compared to nouns, explanations beyond the lexical system across LI groups may lend support to arguments that naming pictures of verbs places different (cognitive) demands on impaired participants than naming nouns (Druks et al., 2006). In the same light, retrieving verb forms from static photographs is more demanding of executive functions, given the unavailability of temporal and movement feature information in a picture—information considered crucial for the recognition of the action in patients with reduced executive resources (d'Honincthun and Pillon, 2005)—which could thus be relevant for all LI groups.

Given the polysemous nature of many verb meanings (e.g., the meaning of the light or general all-purpose verb of *take* differs in “take a book,” “take a break,” or “take a pill”), difficulties in single-word verb retrieval might indeed be expected. With respect to children acquiring Cypriot Greek, this expectation has already been investigated by Grohmann and Leivada (2013) for typical language development as well as Kambanaros and Grohmann (2015) for SLI, something we will return to briefly in the *broad discussion*. Simultaneous activation of more than one lemma for the target action word to evoke selecting the single, correct target verb was difficult for all LI groups. This ties in with reports that, on the one hand, many LI groups have difficulties strategically searching and selecting a word from the lexicon from among many competitors, including individuals with SCZ (Elvevåg et al., 2001), RRMS (Barwood and Murdoch, 2013), and SLI (Mainela-Arnold et al., 2010) and that, on the other hand, verbs are particularly vulnerable (Marvel et al., 2004; Woods et al., 2007). In this case, verbs provoked significantly more semantic circumlocutions or descriptions than nouns for most LI groups (see Table 5 above). In fact, the majority of LI individuals were able to describe something about the verb (i.e., what was happening to the object in the picture), but they were unable to retrieve the form for the target verb. For example, a picture of a woman *sewing* elicited “making needle and thread,” and a picture of a man *drilling* elicited “opening holes with the Black and Decker.” Such responses are appealing, as they show that verb naming is handled differently in Greek, presenting as more diverse in impairment than noun naming.

Regarding the variables affecting naming accuracy, in sum, the RRMS group was different to all other LI groups: Verb (but not noun) naming accuracies were significantly influenced by imageability, word frequency, AoA, and picture complexity. No other LI group showed these effects in total. The SCZ and SLI groups were similar with the RRMS group for verb naming differences because of AoA, while SCZ and RRMS showed similar picture complexity effects for verbs. The SLI group was the only group to show an effect of a psycholinguistic variable on noun naming accuracies, and this was AoA.

What is theoretically important is to accurately profile the naming deficit patterns associated with different clinical conditions. From a clinical perspective, this is actually crucial and may open new strategies for intervention and therapy. Effective language rehabilitation requires a complete detailing of patient strengths and weaknesses. Moreover, patients who share one particular symptom such as a word-retrieval deficit—that is, anomia, characterized as either acquired or developmental—need to be identified. If the present set of ideas is on the right track, the kind of anomia relevant for naming verbs and nouns comes out prominently in confrontation picture naming. The testing and intervention batteries should then be sharpened to target the right kinds of concepts (for some ideas, see also Kambanaros, 2013). Coming back to (A1)–(A3), it is thus possible that the different kinds of semantic errors during word-finding, regardless of grammatical class, in acquired and developmental language impairment are a result of non-focal damage to the neural network representing semantic knowledge, namely the mapping between semantics and the lexicon (Cuetos et al., 2005).

Since (transitive) verb processing requires an understanding of relational concepts, whereas (concrete) nouns are normally non-relational and only need single object reference, this could make verb access and retrieval more complex. In addition, verbs and nouns follow different (semantic) organizational principles: Verbs have been argued to be organized in matrices and nouns in hierarchies (Huttenlocher and Lui, 1979; Kersten and Billman, 1997), implying that a given noun will tend to be strongly related to a small group of other nouns, whereas a given verb will be weakly related to a number of different verbs (for a detailed discussion on both points, see Black and Chiat, 2003; for an alternative conception of a similar idea vis-à-vis first- and second-phase denotations, respectively, see Hinzen and Sheehan, 2014). More research is, however, warranted in relation to grammatical word class processing in LI groups. Considering the diversity of the relationship between word-retrieval deficits and anatomical function, continued study is indeed needed to address the relationship between noun and verb naming difficulties following different LI groups. In the following, we would like to sketch one possible such continuation.

### Broad discussion

A potentially promising, but in any case interesting, route of explanation for such research may be Kayne's (2009) proposal that only nouns are open-class items in the lexicon, whereas verbs should be counted to the closed class—essentially with “nouns being the unmarked category” (Panagiotidis, 2014: 100). Although Kayne's proposal is broadly couched within Phase Theory (Chomsky, 2000 and subsequent work), there is a line of research from outside the generative tradition that seems very much in the same spirit. Going back to at least Gentner (1982), the claim has been made, and substantiated in several psycholinguistic studies, that concrete nouns are conceptually “simpler” than verbs (and that both are more conceptually transparent than function words). In fact, in a series of works, Gentner “proposed that the early priority of nouns in children's vocabularies reflects the fact that nouns are more ‘cognitively dominant’ than verbs and closed-class items” (Fernald and Marchman, 2006, p. 1043), that is, suggesting a scale from fully open- (“cognitive dominance”) to fully closed-class items (“linguistic dominance”), with nouns belonging to the former but verbs being somewhere in between, more toward the closed-class spectrum (e.g., Gentner, 1982; Gentner and Boroditsky, 2001).

The more recent proposal by Kayne (2009), though, follows specific suggestions on phrase structure generation and projection (Chomsky, 2005) as well as valuation processes for formal feature in the syntactic derivation and the question where parametric variation across languages should be expressed (Chomsky, 2001). Kayne defines open-class lexical items by allowing singleton-set formation, having initially valued features, and not being the locus of parametric variation, while closed-class lexical items do allow singleton-set formation, have initially unvalued features, and are the locus of parametric variation. Relevant for us is Kayne's definition of class membership: Only the open-class lexical items are nouns—and, consequently, verbs are closed-class lexical items. In fact, he argues that only nouns can denote (for additional discussion and references, see also Franco et al., 2012), which ties in with Hinzen and Sheehan's (2014) distinction of “first-phase denotations” (nouns) vs. “second-phase denotations” (verbs).

That some verbs would be closed-class lexical items has been suggested for a very long time (at least since Jespersen, 1954), in the form of (semantically) “light verbs,” for example. The particular implementation in modern syntactic theory sees a light verb *v* taking a nominal complement which then combines with *v*. Kayne takes further Hale and Keyser's (1993) proposal: “English *laugh* is a noun that in some sentences co-occurs with a light verb that is unpronounced, giving the (misleading) impression that *laugh* in English can also be a verb. Strictly speaking, though, *laugh* is invariably a noun, even when it incorporates (in some sense of the term) into a (silent) light verb” (Kayne, 2009, p. 336). He essentially suggests that all verbs behave like *laugh*, hence: “All verbs are light verbs” (*ibid*.).

In recent work, we had already set out to capture the difference between the more traditional “light verbs” and the term commonly used in the psycholinguistic literature for a slightly bigger set of verbs, so-called “general all-purpose verbs” (Kambanaros and Grohmann, 2015). What we suggested there is that they share an obvious commonality: Neither verb is fully lexical. We assumed, of course, the classic understanding of verbs and nouns being mostly lexical with the exception of functional light verbs (in the technical sense of Hale and Keyser, 1993 and much subsequent work in generative grammar). However, Kayne's proposal, drastic as it is, may really open up new directions. For example, while there might thus be theory-internal reasons to motivate the suggestion that nouns are the only open-class, i.e., purely lexical, category, it is also a suggestion that can be tested. In a series of works, Franco and colleagues have applied this idea to the verbal behavior of a patient with (logopenic) primary progressive aphasia (e.g., Franco et al., 2012; Franco, 2014), another interesting pathological group that should be included in subsequent research, since it is an acquired language impairment that may affect cognitively and mentally healthy individuals (for noun/verb deficits in a larger group of primary progressive aphasic patients, both agrammatic and logopenic, see Thompson et al., 2012). While producing roughly the same number of sentences as healthy speakers, the participant showed a very low number of lexical verbs in her productions. In contrast, her use of functional verbs (such as modals) and quasi-functional verbs (such as unaccusatives) was normal. As expected, the participant also overused not fully lexical verbs, that is, substituting transitive verbs with light verb constructions, for example, of the sort *fare* “do” plus noun. This kind of data cannot come from picture-naming tasks but need to be collected from speech samples, but a disproportionately better performance on nouns than on verbs in a naming task may be a good sign to probe this further—across pathologies and patients.

To conclude our discussion, we believe that a deeper distinction to be drawn between nouns and verbs in the cognitive-mental lexicon may also offer an interesting twist on Hinzen's (2014) “Un-Cartesian thesis,” the symbiosis of a two-way relationship, perhaps by studying naming to get to words. The classic “Cartesian” take on linguistics (Chomsky, 1966 and much subsequent work in generative grammar) holds “that thought is universal and immutable in [the human] species, while language is merely a contingent way of expressing it in a physical medium” (Hinzen and Sheehan, 2014, p. xvi). Oppose this with “Un-Cartesian” linguistics, the idea “that grammar is the fundamental organizational principle behind a cognitive phenotype that is unique to our species, and defines it” (Hinzen and Sheehan, 2014, p. ix). Then we should not only be able to deduce underlying linguistic principles from the organization of the cognitive system, but also delve deeper into human cognition on the basis of a better understanding of language (see also Chomsky, 2010). To borrow Boeckx's (2013) term, one of the goals of the “comparative biolinguistic” approach is “to uncover the locus of variation (and its constraints) across genotypes, pathologies, or across species” (Leivada, 2014, p. 54; see also Benítez-Burraco and Boeckx, 2014). Looking at grammatical class differences in naming tasks, and finding an overwhelming deficit for verbs as opposed to nouns, across very different pathologies, from developmental language impairment and acquired language disorders to apparently non-linguistic pathologies is one step in this direction.

## Outlook

The study of different language-impaired groups and their performance on word naming tasks offers opportunities for the development of detailed models of cognitive and language functioning. In this article, we explored evidence from several pathological cases and participant groups in the attempt to answer the question whether grammatical class differences play a role in naming within and across different pathological groups. Ultimately, this question bears on the relationship between *thought* (qua cognition) and *language* (qua lexicon), and their linking—even if, we hasten to add, we are not yet in a position to take this step conclusively. But by considering diverse pathologies (as we have investigated here) in different populations (children and adults), even ones that are not primarily related to language (such as schizophrenia or multiple sclerosis), we may in fact learn as much about language as about cognition. We thus side with the assessment that much can be gained from what Hinzen (2014) calls the “Un-Cartesian thesis,” and that such gain will also help with disentangling the issues addressed in the present contribution:

[S]ince no one would want to identify language with a system of pronunciation, and it is clear that language *is* (almost continuously in our waking lives) used internally for purposes of thought as well, in addition to being used for communication, it is a natural suggestion that the cognitive mechanism generating human-specific thought and those generating language should be the same (Hinzen, 2014, p. 227).

Where would we like to head with this research in the future? In closing, we briefly sketch three main areas which we believe are now ripe for further investigation. First, we reported on language-impaired groups that superficially (at least) appear somewhat similar in their naming deficits with the underlying assumption that levels of impairment in word retrieval are comparable across pathologies. What needs to be done next is to expand our evaluation battery and carry out in-depth assessment across a variety of tasks to identify the nature of each individual participant's naming impairment. In the same vein, logistic regression analyses should be performed (i.e., through multiple case study approaches) to confirm the existence of the dissociated naming impairment for verbs and nouns at the individual level across pathologies and decipher the interaction of the naming impairment with primary lexical and semantic word properties. Furthermore, our naming study should be extended to include language-impaired populations with adults and children of the same clinical group (e.g., Down Syndrome) or adults with progressive cognitive impairments (e.g., Alzheimer's dementia). Second, taking our lead from Crepaldi et al. (2014), the GOAT should be standardized using improved statistical modeling that will allow building in sensitivity (i.e., be able to identify language impairment) and specificity indices (i.e., be able to identify non-impairment), perhaps continuing from our earlier work (Michaelides et al., 2011). Third, there now is a better grounded attraction to define how verbs that are conceptually and/or phonologically related to nouns (i.e., the phenomena of instrumentality and name relation, respectively) influence verb naming across language-impaired groups, building on previous research on the topic for individual pathologies such as aphasia (Kambanaros and van Steenbrugge, 2006; Kambanaros, 2009a), SLI (Kambanaros, 2013), and schizophrenia (Kambanaros et al., 2010).

In sum, naming is one possible probe into the relationship between human language and thought. However, in order to be informative, we must have a better grip on what underlies both, the organization of concepts denoting in the real world and leading to the substantive building blocks of human language—ideally, a single, unified mental lexicon. It is our hope, then, that this contribution offers a new angle on interesting data, coming from developing and acquired language impairments as well as patient groups not typically associated with language disorders, and provides the beginnings of novel data analysis. But we also expect that it paves the way for innovative clinical application in the future, especially for patients who also suffer from cognitive and mental health issues. We thus concur with Hinzen and Sheehan (2014, p. xix) that “looking at grammar with Un-Cartesian eyes, therefore, may throw light, not only on thought, but on mental health as well.”

## Author contributions

Both authors contributed to the writing of this article.

### Conflict of interest statement

The authors declare that the research was conducted in the absence of any commercial or financial relationships that could be construed as a potential conflict of interest.
